# Solid-State ^13^C Nuclear Magnetic Resonance Study of Soluble and Insoluble β-Glucans Extracted from *Candida lusitaniae*

**DOI:** 10.3390/molecules28248066

**Published:** 2023-12-13

**Authors:** Ruslan Bikmurzin, Arūnas Maršalka, Lilija Kalėdienė

**Affiliations:** 1 Department of Microbiology and Biotechnology, Institute of Biosciences, Life Sciences Center, Vilnius University, Saulėtekio Ave. 7, LT-10257 Vilnius, Lithuania; 2 Department of Medical Technology and Dietetics, Faculty of Health Care, Vilniaus Kolegija/Higher Education Institution, Didlaukio Str. 45, LT-08303 Vilnius, Lithuania; 3 Institute of Chemical Physics, Faculty of Physics, Vilnius University, Saulėtekio Ave. 3, LT-10257 Vilnius, Lithuania; arunas.marsalka@ff.vu.lt; 4 Nature Research Centre, Akademijos Str. 2, LT-08412 Vilnius, Lithuania

**Keywords:** yeast β-glucan, β-glucan structure, solid-state NMR spectroscopy, yeast cell wall, *Candida*

## Abstract

β-glucans are widely known for their biological activities. However, the choice of extraction method can significantly influence their structural characteristics, thereby potentially impacting their biological functions. In this paper, three fractions of β-glucans were obtained from *Candida lusitaniae* yeast via alkali and hot-water extraction methods and were analyzed using solid-state ^13^C nuclear magnetic resonance (NMR) spectroscopy. Solid-state NMR spectroscopy was used as a nondestructive technique that preserves the structure of the analyzed molecules. The results suggest that differences in the β-glucan structure are affected by the choice of extraction method. The main difference occurred in the 82–92 ppm region with signal presence suggesting that β-glucans have a linear structure when hot-water-extracted, which is absent in alkali-extracted fractions resulting in the acquisition of β-glucans with an ordered, possibly helical structure. A hot-water extracted water-insoluble (HWN) fraction consists of linear β-1,3-glucans with other signals indicating the presence of β-1,6-linked side chains, chitin and small amounts of α-glucan impurities. For those that are alkali-extracted, alkali-insoluble (AN) and water-soluble (AWS) fractions are structurally similar and consist of an ordered β-1,3-glucan structure with β-1,6-linked side chains and a significant amount of α-glucan and chitin in both fractions.

## 1. Introduction

β-glucans are widely known as immunomodulators and are one of the major components of the fungal cell wall. These heterogenous, usually insoluble polysaccharides consist of the highly branched β-1,3-glucan carcass with β-1,6 side chains [1,2,3]. Depending on the source, β-glucan can make 30–80% of the cell wall dry mass, with different degrees and lengths of branching as well as a molecular composition [4,5,6,7,8]. Besides β-glucan, other components such as chitin, mannans, proteins and lipids are cross-connected and important to the maintained structure of the cell wall [1,2,3,9]. These molecules are also abundant in yeast cell walls, including the *Candida* species, with β-1,3-glucan functions as its backbone, cross-linked by chitin with variable lengths of β-1,6-glucan side chains. Together, they form complex structures stabilized by interchain hydrogen bonds and act as linkers for other binding molecules [10,11]. β-glucans play an important role in cellular integrity and are biological response modifiers demonstrating a wide range of activities, including immune response activation through specific receptors. They can act as pathogen-associated molecular patterns (PAMPs), which are important in immune recognition during an infection process [4,8,12,13,14,15]. The structural diversity of β-glucans varies significantly depending on the source of the biological material. However, extraction procedures can result in obtaining different fractions and exhibiting structural variations, including backbone and side chain length, the number of side chains, and the presence of additional molecules. Physicochemical characteristics such as molecular weight and solubility also vary [6,7,8,16,17,18]. As a result, structurally different β-glucan fractions are obtained, which exhibit diverse biological properties [5,15,19] based on the immunomodulatory effect. It is considered that high molecular weight β-glucans with a mass of over 5000 Da exhibit biological activity. By contrast, lower molecular weight β-glucans show limited biological activity or are considered inactive [14,20,21]. Also, it is reported that high molecular weight β-glucans with a molecular weight of 32 kDa per chain have an ordered triple-helical structure. Both are considered the main factors due to their biological activity [22]. Although low molecular weight β-glucans have a random or single-helical structure, some studies find that they also act as immunomodulators [5,8,22]. The solubility of β-glucans is also an important feature, which is mainly affected by the degree and length of β-1,6-glucan side branches and the presence of chitin. Chemical modifications and physical methods can be used to improve solubility by degrading the β-glucan backbone into smaller fragments [23]. The solubility of β-glucans may also act as a limiting factor for their analysis. For example, nuclear magnetic resonance (NMR) spectroscopy in solution requires their solubilization in dimethyl sulfoxide (DMSO), which results in hydrophobic and hydrogen bond destruction. As a result, the denaturation of the triple-helical structure occurs, and random coils or single helices are formed [22,24]. However, analytical techniques, such as Fourier transform infrared (FT-IR) and solid-state NMR (ssNMR) spectroscopy allow for the analysis of the preserved native structure and high molecular weight β-glucans, which circumvents the problems of insoluble β-glucans analysis [2,3,25,26]. Therefore, ssNMR spectroscopy, was used to compare the alkali-extracted water-soluble (AWS) and alkali-insoluble (AN) as well as hot-water-extracted and water-insoluble fractions (HWN) of β-glucans from *Candida lusitaniae.*

The isolation of β-glucan is a complex, multi-step, time-consuming process. Different extraction and drying methods can also have an impact on the glucan structure, which may result in different biological effects [8,19,20]. Depending on the purpose of the research, various fractions are used as follows: β-glucans, the β-glucan–chitin complex, α-glucan, zymosan, and other molecules. Thus, different isolation and purification techniques are required. Solid-state nuclear magnetic resonance spectroscopy has gained more interest in β-glucan analysis. This nondestructive technique effectively maintains the three-dimensional structure of the analyzed polymer without interference from solvents [2,3,25,27]. Some of the key chemical shifts attributed to β-glucans and other molecules can reveal information about their composition and conformation. The C1 carbon signal in the range of 102–105 ppm is indicative of β-1,3-glucans, C3 characteristic signals in a range of 82–92 ppm possess information about conformation, and C6 carbon signals in a 55–65 ppm range allow interconnections to be distinguished between β-1,3-glucans, β-1,6-glucans and chitin [2,17,24]. In this paper, several fractions of β-glucans were obtained using the following two popular methods: alkali and hot-water extraction. The aim of the present study was to compare soluble and insoluble β-glucans extracted from *Candida lusitaniae* using solid-state NMR spectroscopy.

## 2. Results

Three fractions of β-glucans were extracted from the yeast *C. lusitaniae*. Alkali-insoluble (AN, also water-insoluble) and water-soluble (AWS, also alkali-soluble) fractions were obtained via alkali extraction. The third fraction of water-insoluble (HWN) β-glucan was obtained using the hot-water extraction method.

**Figure 1:**
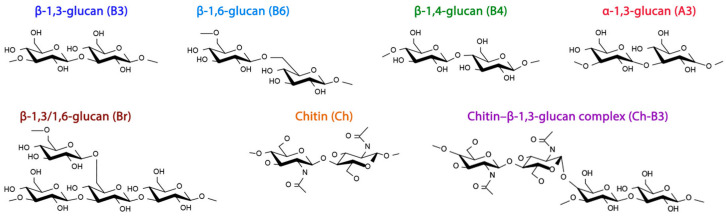
Skeletal structure of glucans and chitin.

**Figure 2:**
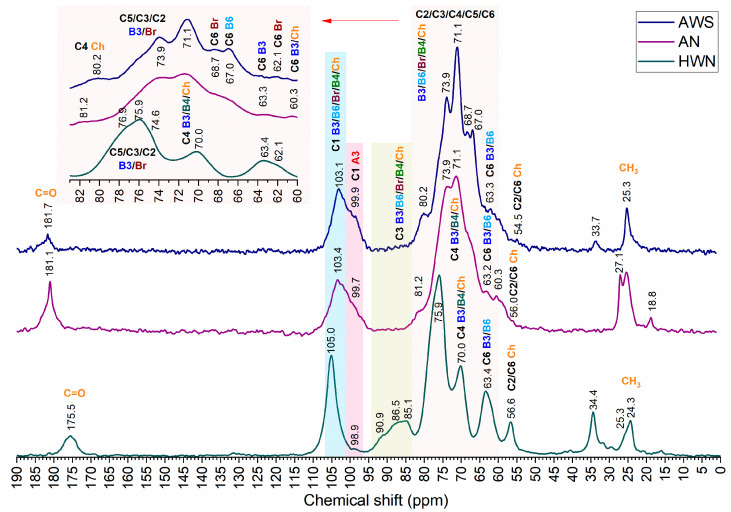
The normalized ^13^C MAS NMR chemical shifts of alkali-extracted alkali-insoluble (purple) and water-soluble (blue) fractions and the hot-water-extracted water-insoluble (green) β-glucan fraction. The 83–60 ppm region is enlarged in the pink rectangle.

**Figure 3:**
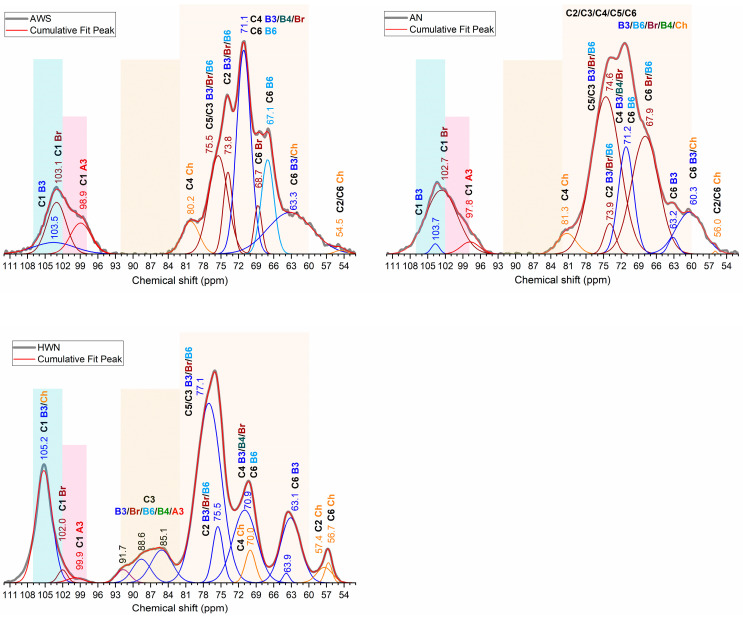
^13^C MAS NMR spectra of the 54–110 ppm region where band deconvolution with the application of Gaussian curve-fitting was applied. Fractions from the top left: AWS, AN and HWN.

The obtained fractions contained β-glucans with mixed linkages, including α-glucan and chitin (Figure 1 and Figure 2). The overlapping of C1–C6 carbon signals from these molecules poses difficulties in data analysis and interpretation. However, using NMR spectra deconvolution, we could partially reveal some hidden peaks and simulate individual C1–C6 carbon peak resonances in the range of 54–110 ppm (Figure 3). Slight differences in the chemical shift between normalized and deconvoluted spectra occurred but did not exceed 0.2 ppm. It is also important to note that signal intensity variation and the shifting of different fractions are affected by sample hydration, including differences in molecular weight and the overlapping of signals from different molecules, e.g., C3 of β-glucans and α-glucans and C4 and C6 from β-glucans and chitin in the characteristic regions [3,24,28]. Although severe overlapping does not allow detailed analysis, the simulation of C1–C6 carbon peaks of interest, attributed to β-glucans, were achieved with good precision based on R^2^ and RMSE values. The R^2^ values of 0.9851, 0.9300, 0.9676 and RMSE values of 0.0169, 0.0438 and 0.0249 were achieved for AWS, AN and HWN, respectively, indicating a good fit [29].

Chemical shift assignments for individual C1–C6 β-glucan and chitin carbons were based on an analysis of the literature and the deconvolution of obtained spectra, which are summarized in Table 1. The chemical shifts of C1, C2, C3, C4, C5 and C6 carbons from the deconvoluted HWN fraction spectrum were assigned at 105.2, 75.5, 88.6, 70.9, 77.1 and 63.1 ppm, respectively. These are indicative of linear β-1,3-glucans. However, additional C4 and C6 carbon signals of branched β-1,3/1,6-glucans and β-1,6-linked side chains could also be distinguished, as well as α-glucan and chitin peaks (Figure 2 and Figure 3; Table 1). In comparison, the fractions of AN and AWS represented mainly branched β-glucans with limited signal intensity from β-1,3-glucan. In the AN deconvoluted spectrum, chemical shifts were assigned at 102.7, 73.9, 71.2, 74.6 and 67.9 ppm and corresponded to C1, C2, C4, C5/C3 and C6, respectively. The chemical shifts of C1, C2, C4, C5/C3 and C6 carbons from the deconvoluted AWS spectrum were observed at 103.1, 73.8, 71.1, 75.5 and 68.7 ppm, corresponding to C1, C2, C4, C5/C3 and C6 carbons, respectively [2,12,16,17,30,31,32]. Unfortunately, in alkali-extracted fractions, we were unable to deconvolute the separate C3 carbon signal at a statistically significant level due to severe overlapping with the C5 signal [17,32].

The main differences in the obtained fractions were observed at C1 characteristic signals of β- and α-glucans in the respective regions of 102–105 ppm and 99–101 ppm, a C3 characteristic broad signal in the range of 82–92 ppm, C6 carbon and chitin signals in the 55–65 ppm range [2]. The C1 signal of β-and α-glucans additionally overlapped with the signal from chitin. The heterogeneity of β-glucans, or, in other words, the connection of β-glucans with other molecules, can result in a broader C1 signal. Conformation might also impact the signal pattern, e.g., the narrower signal with a higher chemical shift is indicative of a conformation similar to β-chitin [15,19,33].

The HWN fraction exhibits a strong and narrow C1 signal, which is mainly attributed to β-1,3-glucans with a low number of β-1,6-linked side chains and chitin connections. Branched β-1,3/1,6-glucans in this fraction exhibit lower intensity alongside the chemical shift in the C1 signal at 102.0 ppm. The low intensity, broad signal of α-glucan C1 carbon is observed at 99.9 ppm, indicating its small amount in this fraction. AN and AWS fractions also exhibit three different C1 signals. The predominant signal is attributed to branched β-1,3/1,6-glucans. Signals with a chemical shift of 103.5 ppm in the AWS fraction and 103.7 in the AN fraction are attributed to linear β-1,3-glucans. In the AN fraction, a narrow, low-intensity signal is found, indicating low levels of homogenic β-1,3-glucans [2,25,27,30,34]. By contrast, in the AWS fraction, β-1,3-glucans are heterogenic and possibly connected to other molecules, e.g., α-glucans. The significant intensity of α-glucan-characteristic C1-anomeric carbon resonance is observed in AWS at 98.9 ppm and in AN at 97.8 ppm. In fact, in the AWS fraction, resonance is the strongest, indicating the highest content amongst different fractions [1,2,19,26].

The C3 characteristic signal at 82–92 ppm is related to linear β-1,3-linked residues [17,26,28] and is only observed in the HWN fraction. The lack of this signal in alkali-extracted β-glucan fractions suggests the ordered state of the β-1,3-glucan backbone, which is probably a triple helix maintained by intra- and intermolecular hydrogen bonds [2,10,22,23] or a double helix with extensive hydrophobic interactions involved in their structure [24,28,35]. Similar findings were made by Pramod K. Gopal [36] and other researchers [23,24], who reported the branched β-1,3-glucan structure with β-1,3- and β-1,6-side chains alongside an absence of the ^13^C-signal for β-1,3-linkages.

C1 carbon signals of β-glucans together with 67.1 and 67.9 ppm in AWS and AN fractions, respectively, are possibly attributed to additional connections between C1 and C6, giving a cross-peak signal at the C6 position [2,25]. Another peak at 68.7 ppm is probably an overlapping peak of β-1,4-linked chitin C2 carbon and C6 carbons of branched β-1,3/1,6-glucans [2,28]. Other C6 carbon signals of β-1,3-glucan and chitin are located near 63 and 60 ppm, respectively [34]. In the AN fraction, the deconvolution of this signal could not be simulated at a significant level, but due to the similarity of alkali-extracted fractions, we assumed its presence. In the HWN fraction, a similar signal at 63.1 is attributed only to β-1,3-glucan. The carbon signal at around 71 ppm overlaps with the C4 carbon mainly from β-1,3-glucans with several other signals. It could also be attributed to C4 from chitin and β-1,4-glucan [19,24,32] as well as the O-substituted C6 of β-1,6-glucan [28] or the overlapping of C3 carbon with β-1,4-linked chitin and the C4 carbon of β-1,6-glucan [2,28,32]. In the HWN fraction, deconvolution revealed the following two separate peaks: 70.9 attributed to C6 of β-1,6- and branched β-1,3-glucans, and 70.0 ppm attributed to C4 resonance involved in β-1,4-linkage [19,24,32]. Differences in signal intensity and signal width could be attributed to the composition differences of the obtained fractions. The data indicate the presence of chitin and β-1,6-side chains in alkali-extracted fractions with a higher relative β-1,6-glucan content in the AWS fraction compared to AN and the lowest content in the hot-water-extracted fraction.

Chitin seems to be more prevalent in the HWN fraction, with lower content in the AN and AWS fractions. Chitin characteristic signals are observed near 24, 34, 130 and 174 ppm [12]. Similar signals of the carbonyl group (C=O) are observed at 175.5 ppm in HWN, 181.1 ppm in AN and 181.7 ppm in AWS fractions. The signal from the methyl group (CH_3_) is present at 25.3 ppm in all fractions. Other signals, for example, the resonance at 80.2 ppm in AWS and 81.2 ppm in AN, could be attributed to the C4 involved in β-1,4-linkage between chitin and β-1,3-glucan [32,37]. Additionally, resonance at 57.4/56.7 ppm in HWN, at 54.5 ppm in AWS and 56.0 ppm in AN can be attributed to the acetyl group of C2 carbon and C6 carbon [2,19,26,38,39].

The differences in the ^13^C NMR spectra of obtained β-glucans (Figure 1) indicated the connection between the isolation method and the structural variation in the obtained molecules. Different methods utilize diverse processes during the extraction. Different processing results are obtained for the structural and compositional differences of β-glucan fractions.

## 3. Discussion

Linear β-glucans can be distinguished from branched β-glucans due to the differences in characteristic C1, C3 and other carbon signals in a range of 60–80 ppm, which are attributed to β-1,3/1,6-glucans, as well as β-1,4-linked chitin and α-glucan [14,18,19,24,37]. The chemical shifts of C1–C6 carbons in the HWN fraction indicate mainly β-1,3-glucan backbone with β-1,6-glucan and chitin interconnections [17,32]. The differences in C1 chemical shift values depend on interconnections between β-glucans and other molecules [26]. A higher number of β-1,6-glucan side chains or β-1,4-glucans could result in the C1 signal shift toward lower values. However, the β-1,3-glucan–chitin complex results in higher C1 chemical shift values [25,26]. Sample hydration and molecular weight could impact the chemical shift with lower water content, resulting in a chemical shift toward higher values [26,40]. In the HWN fraction, higher chemical shift values could be attributed to →3)-β-Glcp-(1→ linkage and the β-linked non-reducing ends of β-Glcp-1→. On the contrary, lower C1 chemical shift values in AWS and AN fractions could be explained by the presence of →3,6)-β-Glcp-(1→ linkage, which could be formed by reducing the end of β-1,6-glucan connection to the non-reducing terminal glucose of β-1,3-glucan [17,24,40,41]. This, in turn, could explain the absence of the C3 characteristic signal related to β-1,3-linked residues between 82 and 92 ppm [17,26,28] in AWS and AN fractions and its presence in the HWN fraction. 

β-1,3/1,6-glucans are proposed to form the rigid network of the fungal cell wall with β-1,3-glucan as a core component of the yeast cell wall, making up 30% of the dry mass of the yeast cell [2,24,27]. Although β-glucan interconnections with other cell wall components are still poorly understood [4], two research groups found that α-1,3-glucan together with chitin might form the rigid hydrophobic core instead of multi-branched β-1,3/1,6-glucans in *Aspergillus fumigatus* [1,2,19] and *Schizophyllum commune* [25]. New insights on the structure of the cell wall of *Aspergillus fumigatus* and *Schizophyllum commune* revealed that the core of the cell wall consists of β-glucan, mannan and chitin held together by covalent bonds [1,2,19,25]. The core of *Candida albicans’* cell wall is also made of chitin and the β-1,3–glucan complex [10] through β-1,4-glycosidic linkage, making it insoluble [12,25]. Solubility also depends on the molecular weight of the polymer and its branching; the more branched and heavier β-glucan is, the more it is solubilized [12,18]. The branching of the 3,6-linked glucose residue facilitates the binding of other cell wall components, such as mannoproteins and chitin, making β-1,3-glucan essential in maintaining crosslinking and branching in the cell wall [1,4,10,18].

Chitin and α-glucan characteristic signals are present in all fractions, which is not unusual for β-glucans. These molecules can possess mixed α-linkages in different carbon linkages [26]. The peaks of the carbonyl group and aliphatic carbons are also attributed to the presence of some proteins. Signals from the aromatic region 120–140 ppm are inconclusive. Thus, the singular presence of a peak around 33 ppm could suggest the presence of specific non-aromatic amino acids [2,3,27,42]. However, the cross-connections between glucans, chitin and proteins must be established [25]. Peaks at 34.4 ppm could also be the traces of fatty acids or other compounds that remain after the extraction process [1,43,44,45,46]. The upshift of the carbonyl group signal in the AN and AWS fractions, compared to HWN, might appear due to chemical modification, structural changes or even the degradation of chitin or exposure of additional molecules connected to β-glucan [25]. Also, the presence of other sugars would explain some chemical shift variations in our samples [27]. Interestingly, the alkali-insoluble fraction lacks this signal, and the soluble fraction has a low-intensity peak. Although usually the 20–35 ppm region is attributed to the presence of lipids, the peak near 24 ppm also represents the methyl group of the chitin or methylene group in melanin. Researchers showed that fungal melanin has a strong resonance near 30 ppm and near 175 ppm [45,46]. We cannot rule out the presence of the chitin–melanin complex due to the hydrophobic nature of melanin [45,47,48] and the yellowish-brownish color of the obtained β-glucan fractions, which is clearly due to the presence of pigments. At this point, we cannot provide a more detailed analysis and explanation. Thus, the origin of the protein, lipid and/or melanin presence and connection to β-glucans are the questions of further research.

Glycogen, an α-1,4-glucan used to store glucose, is often co-isolated from yeast together with β-glucans [6,12]. It was reported that the cell wall of several *Candida* species, *C. albicans*, *C. dubliniensis*, *C. haemulonii* and *C. auris*, has an unusual complex of glycogen covalently linked to β-1,3/1,6-glucans [12], which could explain the appearance of α-glucan signals in our samples from *Candida lusitaniae*. The key peak signal from the C1 carbon of α-glucans is observed at 98–101 ppm and has a noticeable difference in alkali and hot-water extracted fractions. Another α-glucan key peak of C2/5 carbon is near 72 ppm. However, they strongly overlap with other signals from β-glucans and chitin [44]. The broad signal in the 82–92 ppm region consists of overlapping signals of C3 carbon from α-glucans, β-glucans and chitin due to exposed terminal residues. In the AWS and AN fraction, this signal is absent [19,26] which might suggest that glycogen forms a complex with β-glucans and chitin. Similarly, D.W. Lowman and colleagues concluded that the majority of the β-1,3/1,6-glucans in the *C. albicans* cell wall exists as a macromolecular complex with glycogen [12]. Additionally, it was found that α-1,3-glucan in *A. fumigatus* cross peaks with chitin plays an important role in cell wall organization and is mainly extracted by hot alkali [1,2,19]. Interestingly, it was shown that samples without chitin had a higher content of α-1,3-glucan [19]. Hot-water-extracted β-glucans possibly contain more chitin compared to alkali-extracted fractions; however, this needs confirmation using other methods. Differences in extraction procedures could affect the content of glycogen in different fractions; however, we cannot exclude the possible presence of α-1,3-glucan in the obtained β-glucan fractions. Further investigation is necessary to acquire additional details.

Alkali and hot-water extraction results were obtained from conformationally and compositionally different β-glucan fractions. These variations could be explained by several hypotheses. One possibility is that α-glucan plays a role in maintaining the helical structure of β-glucans. However, it is also plausible that the HWN fraction might contain β-glucans in a triple-helical state. β-glucans obtained via hot-water extraction could potentially retain higher amounts of cross-linked β-1,6-linked and β-1,3-linked side chains along with chitin. This would also explain the higher molecular weight of this fraction and the C3 signal appearance in the 82–92 ppm region. On the other hand, hot-water extraction might disrupt the intramolecular bonds involved in the formation of an ordered β-glucan structure and the less efficient breakage of glycosidic bonds, exposing terminal residues. This disruption could lead to the less efficient breakage of glycosidic bonds, thereby exposing the terminal residues. These hypotheses are the subjects for future research.

## 4. Materials and Methods

### 4.1. Yeast Strain and Culture

*Candida lusitaniae* yeasts were kindly provided by Vilnius University LSC of the Microbiology and Biotechnology Department.

Yeast cells were inoculated into an Erlenmeyer flask containing 500 mL of the liquid YPD culture medium and were grown for 48 h at 37 °C, with gentle shaking at 160 rpm. Biomass was collected via centrifugation at 5000× *g* for 5 min at 4 °C and washed three times with distilled water [49].

### 4.2. YeastAautolysis

The β-glucan extraction was carried out using the alkali and hot-water extraction methods. In both cases, the first step was to remove yeast intracellular components by performing autolysis in distilled water at a ratio of 1:20 (*w*/*v*) with 3% of sodium chloride added for 24 h at 60 °C with 120 rpm of agitation in an orbital shaker. Afterward, the autolysate was incubated at 80 °C for 15 min in a water bath to deactivate endogenous enzymes. Then, autolyzed cell walls were collected via centrifugation at 5000× *g* for 10 min. The supernatant was discarded, sediments were washed three times with distilled water and air-dried at 60 °C until dry [49,50,51,52]. Dried cell walls were divided into two groups and subjected to further isolation by hot water and alkali methods.

### 4.3. Hot-Water Extraction

β-glucan extraction using hot water was carried out using the modified method originally described by Liu et al. [50] and described in our previous work [42]. Briefly, cell wall preparations were suspended in distilled water and autoclaved at 121 °C for 4 h. After this, suspension was centrifuged at 5000× *g* for 10 min, the supernatant was discarded, cell walls were washed three times with distilled water and air-dried at 60 °C until dry. The obtained fraction of the autoclaved cell walls was further suspended in 50 mL of distilled water at a 10:1 (*v*/*w*) distilled water to cell wall ratio and subjected to sonication in an ice bath. Sonication was performed at 40% of the maximum output with a 20 kHz frequency and power of 240 W. Disruption consisted of 30:10 s (on: off) cycles for 15 min [50,52,53]. After sonication, degraded cell walls were centrifuged and washed three times with distilled water at 5000× *g* for 5 min at 4 °C. Obtained sediments were treated with isopropyl alcohol at a ratio of 1:4 (*w*/*v*) for 2 h at 80 °C using a magnetic stirrer. Pellets were collected via centrifugation at 7000× *g* for 10 min at 4 °C, washed three times with acetone and 5 times with distilled water. The obtained β-glucan fraction was named HWN, dried in a hot air oven at 60 °C, powdered and subjected to ^13^C NMR analysis [50,52,53,54].

### 4.4. Alkali Extraction

Cell wall preparations obtained after autolysis (Section 4.2) were suspended in 50 mL of distilled water in a 10:1 (*v*/*w*) distilled water to cell wall ratio and subjected to disruption via sonication in an ice bath using an ultrasound. Sonication was carried out as described previously with time, and the on/off ratio was changed to 30 min and 45:15 s, respectively.

After sonication disrupted the cell walls, they were washed three times. Intact cell walls were subjected to an additional round of sonication [54]. The obtained pellets of degraded cell walls were further subjected to alkali treatment with 1 M NaOH for 4 h at 90 °C. Sediments after alkali treatment, containing insoluble β-glucans, were collected via centrifugation at 5000× *g* at 4 °C for 10 min and washed three times with distilled water [51,52,54]. The obtained fraction named AN was dried in a hot air oven at 60 °C, powdered and subjected to ^13^C NMR analysis.

The supernatant was produced by obtaining sediments after alkali treatment possessing the fraction of water-soluble β-glucans. The supernatant was neutralized with 2 M of acetic acid, centrifuged, and produced protein sediments before being discarded. Water-soluble β-glucans were precipitated from the obtained supernatant with three volumes of ethanol [8,54]. Precipitated β-glucans were centrifuged, air-dried in a hot air oven at 60 °C, powdered and subjected to ^13^C NMR analysis as a fraction of water-soluble β-glucans named AWS.

### 4.5. Solid-State Nuclear Magnetic Resonance

NMR measurements were carried out on a Bruker AVANCE III HD spectrometer (Bruker Biospin GMBH, Ettlingen, Germany) operating at the resonance frequencies of 400 MHz for ^1^H. The experiments were performed in a 9.4 T magnetic field using an Ascend-wide bore-superconducting magnet. All MASs (magic angle spinning) measurements were performed using a Bruker 4 mm H/X CP-MAS probe-head. NMR MAS measurements were performed at the spinning rate of 10 kHz using a 4 mm zirconia rotor at a 300 K temperature. The Larmor frequency for ^13^C was 100.62 MHz, and chemical shifts were referenced to adamantane. ^13^C MAS spectra were accumulated using 1024 scans with a repetition delay of 3 s. A rectangular variable contact time pulse (1 ms) for ^13^C and a ramped 50–100% pulse for ^1^H were used in CP MAS experiments in order to fulfill one of Hartmann–Hahn’s matching conditions. 

### 4.6. Spectral Analysis, Deconvolution and Curve-Fitting

NMR spectra were processed using Topsin 3.2 software. The obtained data were then transferred and further analyzed using OriginPro^®^ 2023b (Learning Edition) software.

Spectra were normalized, and baseline correction was performed using the second derivatives with additional points added manually, which were smoothed using the adjacency averaging method (measurement smoothing window 3). Afterward, the deconvolution of the 54–110 ppm region of obtained spectra, containing bands relating to C1–C6 sugar carbons, was performed.

In order to deconvolute the overlapping bands in this region and accurately determine the individual bands, the Levenberg–Marquardt algorithm with nonlinear Gaussian and Voigt curve-fitting functions was employed. The main peaks were picked manually, maintaining the minimal number required for the convergence and a good fit. The fit of Voigt curves (which is a convolution of a Gaussian function and a Lorentzian function) did not converge; thus, only Gaussian curve-fitting was used further. Gaussian peaks were added in the positions corresponding to the most prominent resonances, and band assignments were performed based on an analysis of the literature. The spectra were deconvoluted using Gaussian curves and a constant baseline (constrained to zero) [55,56,57]. The fitting was iterated until convergence, and a Chi-Square tolerance value of 10^−9^ was obtained. The R^2^ and RMSE (root-mean-square error) values from the summary of curve-fitting were used as indicators for goodness of fit, with higher R^2^ and lower RMSE values indicating a good fit [29].

## 5. Conclusions

In this work, using solid-state ^13^C NMR spectroscopy, we evaluated the structural differences of β-glucans extracted from *Candida lusitaniae* by alkali and hot water. Different extraction methods resulted in the structural and compositional variation in the obtained β-glucan fractions, which could be traced through chemical shift changes in the spectra. The deconvolution of solid-state ^13^C NMR spectra is useful in order to find hidden peaks; however, regions with severe overlapping, e.g., C2/C3/C5, are still hard to analyze. C3 carbon produces a characteristic broad signal in the 82–92 ppm region due to β-glycosyl terminal residues, indicating the linear structure of the β-1,3-glucans. However, the absence of this signal is indicative of an ordered, helical structure of β-1,3-glucans. The hot-water-extracted fraction of water-insoluble β-glucans exhibits the linear structure of β-1,3/1,6-glucans and chitin with a small amount of α-glucan impurities. Alkali extraction allows structurally similar alkali-insoluble and water-soluble fractions to be obtained. Both fractions exhibit an ordered β-1,3-glucan structure with β-1,6-linked side chains and a significant amount of α-glucan and chitin. α-glucan is represented by glycogen and found in the cell walls of several *Candida* species. It is possible that other sugars are also present in alkali-extracted fractions due to the unusual shift of the carbonyl signal near 180 ppm. This might be due to structural changes in the chitin–glucan complex or possibly the appearance of other β-glucan-bound molecules. All fractions might have non-aromatic amino acids and lipid or melanin impurities. The solid-state NMR study has the advantage of revealing the preserved structure of the analyzed molecules. An analysis in solid-state does not require the prior preparation of the sample; however, some parts of the spectrum can be difficult to interpret due to signal overlapping. Partially, this can be overcome using signal processing via deconvolution. Still, ^13^C ssNMR is a powerful method that reveals small details of the extracted β-glucan fractions, although the method of choice for the structural analysis of β-glucans depends on the goal of the research.

In the end, alkali and hot-water extracted β-glucans exhibit structural and compositional variations that may affect their biological activity. It is important to maintain consistency in all the aspects of the experiments with β-glucans. Thus, it is important to choose isolation techniques and purification methods for β-glucans since they may affect the outcome of the biological experiments.

## Figures and Tables

**Table 1:** ^13^C NMR individual carbon shifts of β-glucans and chitin from different fractions.

Carbon Assignment	Fraction	C1	C2	C3	C4	C5	C6	CH_3_	C=O
β-1,3-glucans	AN	103.7	73.9	n.d. *	71.2	74.6	63.2		
	AWS	103.5	73.8	n.d. *	71.1	75.5	63.3		
	HWN	105.2	75.5	82–92	70.9	77.1	63.1		
β-1,3/1,6-glucans	AN	102.7	ov. **				67.9		
	AWS	103.1					68.7		
	HWN	102.0					70.9		
Chitin	AN	ov. **	56.0	ov. **	81.3	ov. **	60.3	25.3	181.1
	AWS	ov. **	54.5	ov. **	80.2	ov. **	60.3	25.3	181.7
	HWN	ov. **	57.4	ov. **	70.0	ov. **	56.7	24.3	175.5

* n.d.–no data; ** -ov.–overlapping with the corresponding region of β-glucan.

## Data Availability

Data are contained within the article.
